# Revealing biophysical properties of KfrA-type proteins as a novel class of cytoskeletal, coiled-coil plasmid-encoded proteins

**DOI:** 10.1186/s12866-020-02079-w

**Published:** 2021-01-22

**Authors:** M. Adamczyk, E. Lewicka, R. Szatkowska, H. Nieznanska, J. Ludwiczak, M. Jasiński, S. Dunin-Horkawicz, E. Sitkiewicz, B. Swiderska, G. Goch, G. Jagura-Burdzy

**Affiliations:** 1 Warsaw University of Technology, Faculty of Chemistry, Chair of Drug and Cosmetics Biotechnology, Noakowskiego 3, 00-664 Warsaw, Poland; 2 Department of Microbial Biochemistry, Institute of Biochemistry and Biophysics PAS, Pawinskiego 5a, 02-106 Warsaw, Poland; 3 Nencki Institute of Experimental Biology PAS, Laboratory of Electron Microscopy, Pasteura 3, 02-093 Warsaw, Poland; 4 University of Warsaw, Centre of New Technologies, Laboratory of Structural Bioinformatics, 02-097 Warsaw, Poland; 5 Nencki Institute of Experimental Biology, Laboratory of Bioinformatics, Pasteura 3, 02-093 Warsaw, Poland; 6 Mass Spectrometry Laboratory, Institute of Biochemistry and Biophysics, Polish Academy of Sciences, Pawinskiego 5a, 02-106 Warsaw, Poland

**Keywords:** Broad-host-range plasmids, Self-assembly, Coiled-coil proteins, DNA-protein interaction, Stability functions, Brownian motion

## Abstract

**Background:**

DNA binding KfrA-type proteins of broad-host-range bacterial plasmids belonging to IncP-1 and IncU incompatibility groups are characterized by globular N-terminal head domains and long alpha-helical coiled-coil tails. They have been shown to act as transcriptional auto-regulators.

**Results:**

This study was focused on two members of the growing family of KfrA-type proteins encoded by the broad-host-range plasmids, R751 of IncP-1β and RA3 of IncU groups. Comparative in vitro and in silico studies on KfrA_R751_ and KfrA_RA3_ confirmed their similar biophysical properties despite low conservation of the amino acid sequences. They form a wide range of oligomeric forms in vitro and, in the presence of their cognate DNA binding sites, they polymerize into the higher order filaments visualized as “threads” by negative staining electron microscopy. The studies revealed also temperature-dependent changes in the coiled-coil segment of KfrA proteins that is involved in the stabilization of dimers required for DNA interactions.

**Conclusion:**

KfrA_R751_ and KfrA_RA3_ are structural homologues. We postulate that KfrA type proteins have moonlighting activity. They not only act as transcriptional auto-regulators but form cytoskeletal structures, which might facilitate plasmid DNA delivery and positioning in the cells before cell division, involving thermal energy.

**Supplementary Information:**

The online version contains supplementary material available at 10.1186/s12866-020-02079-w.

## Background

Research over the last two decades has revealed that bacterial cells are surprisingly complex in their architecture. The homologs of three major eukaryotic classes of cytoskeletal elements, actins, tubulins and intermediate filaments (IFs), have been discovered in bacteria and their biological functions have been assigned [1]. The proteins play a role in a variety of processes, including morphogenesis (e.g. actin-like MreB and IF-like crescentin, [2, 3], cytokinesis (tubulin FtsZ and actin FtsA-ring formation) [4], DNA segregation (plasmid-encoded actin-like ParM and AlfA, tubulin-like TubZ) and organelles segregation (actin-like MamK) [5, 6]. Besides that, a variety of alpha-helical coiled-coil proteins (long rod-like or segmented) were discovered to be able to assemble into filaments in the nucleotide dependent or in an independent manner [7]. Coiled-coil proteins are assemblies of two or more alpha-helices that are packed together in a parallel or anti-parallel fashion [8]. The general feature of coiled-coil domains appears to be their ability to act as “cellular velcro” to hold together molecules or subcellular structures. They provide cables and networks in the cytoskeleton, molecular scaffolds for other proteins e.g. ZapB in FtsZ-ring formation [9], and are involved in regulation of transcription. When combined with ATPase or GTPase domains they often function in protein folding (chaperonins) or DNA remodeling (SMC, MukB, nucleases or topoisomerases).

KfrA proteins were first identified as encoded by a low-copy number broad host range plasmids (BHR) capable of replication and stable inheritance in a variety of Gram-negative [10–12] and Gram-positive bacteria [13] although with no implications about their possible functions. Even though KfrA proteins of IncP-1 plasmids, RK2 and R751, have been postulated to be involved in the stable maintenance of cognate plasmids [10, 11], the molecular mechanism of KfrA action and their exact biological role remain unclear. The biophysical and biochemical studies on KfrA_RK2_ protein encoded by representative plasmid of IncP-1α group have shown, that it has an unusually high alpha-helical content [11]. The predicted globular head of KfrA_RK2_ (N-KfrA domain) was involved in the specific DNA binding activity, whereas alpha-helical tail, with no assigned function, was similar to chemo-mechanical force-generating proteins like myosin and SMC (Structural Maintenance of Chromosome) proteins, ubiquitous in the living world [14, 15]. SMC proteins are anti-parallel dimeric factors crucial for chromosomes’ condensation and cohesion, gene regulation and DNA repair in eukaryotic organisms. Prokaryotic SMC/MukB complexes [16–19] are involved in condensation and organization of the chromosomes and their impairment leads to the visible defects in chromosome segregation (reviewed by [20].

**Fig. 1:**
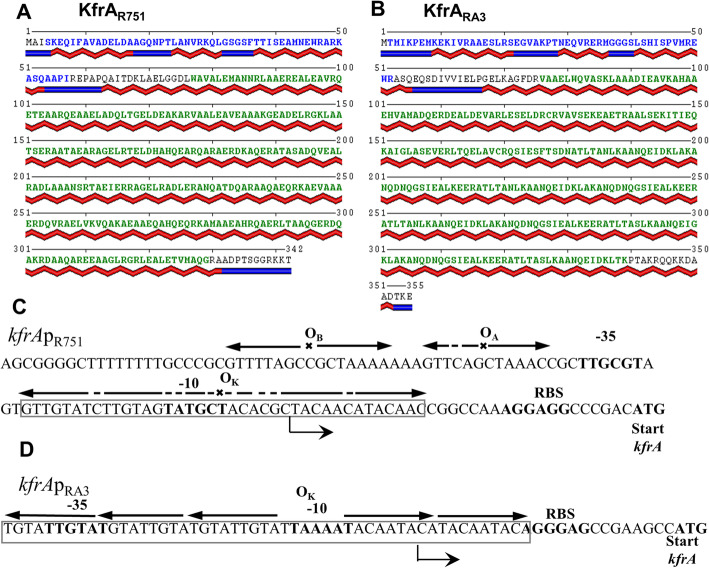
Predicted structures of KfrA proteins from R751 and RA3 plasmids and organization of *kfrAp* regions. KfrA_R751_ (IncP-1β) (**a**) and KfrA_RA3_ (IncU) (**b**) amino acid sequences predicted secondary structure visualized with POLYVIEW-2D server. Sequence regions corresponding to the HTH and coiled-coil domains are marked in blue and green, respectively. The secondary structure is shown below the sequence as red ribbons (helices) and blue bars (strands). **c** *kfrA*_R751_ promoter region with − 35 and − 10 motifs indicated in bold. Start codon and rbs **–** ribosome binding site for KfrA and binding sites for regulatory proteins are shown: O_A_- KorA_R751_ operator, O_B_- KorB_R751_ operator, O_K_- putative KfrA _R751_ operator. **d** *kfrA*_RA3_ promoter region with − 35 and − 10 motifs indicated as above. Putative KfrA _RA3_ binding site (O_K_) of five 9 nt repeats is indicated by arrows

KfrA proteins, which are the members of a large family of plasmid encoded proteins, show low similarity in the primary amino acids sequence, however, they are predicted to be almost 100% alpha helical [10–13, 21–24]. Their role as the accessory proteins in plasmid segregation [10, 11] may result from formation of alpha-helical filaments interacting with the bacterial host scaffold or engagement, as a SMC-like complex, in condensing plasmid DNA to an optimal structure and facilitating its movements through the dense cytoplasm matrix to gain proper positioning. In this study we focused on biophysical properties of two KfrA proteins, encoded by BHR plasmids of IncP-1β subgroup and IncU incompatibility groups, R751 and RA3, respectively (Fig. 1a-b). The *kfrA*_*R751*_ and *kfrA*_*RA3*_ genes are encoded in proximity to the *incC* and *korB* operon encoding Type I active partition proteins [25]. Both KfrAs are site-specific DNA binding proteins, which auto regulate cognate operon expression in vivo [10, 26] similarly to the archetype KfrA of RK2 plasmid [11]. The putative target for KfrA_R751_ is assumed to be an extended inverted repeat overlapping the − 10 region of the *kfrA*p_R751_ tentatively named KfrA_R751_ operator O_K_ [10] (Fig. 1c). The putative target for KfrA_RA3_ consists of five 9 bp iterations overlapping the − 10 region of the *kfrA*p_RA3_ [12] (Fig. 1d).

It was found that the helix-turn-helix (HTH) DNA binding motif is localized in the N-terminal part of KfrA_R751_, whereas the alpha-helical domain may contribute to the stabilization of the active form of the protein as a transcriptional regulator [10]. The dimerization of KfrA_R751_ protein has been demonstrated experimentally in the yeast two-hybrid system [10]. The analysis of data provided by atomic force microscopy (AFM), revealed that in solution, the major form of KfrA_R751_ protein was a tetramer in the hexagonal configuration, resulting in an elongated shape of tetrameric protein complex [27]. Despite low conservation of the primary sequences, KfrA_R751_ and KfrA_RA3_, seemed to share the secondary structure, (Fig. 1a-b), however, this has not yet been verified experimentally. In this work, circular dichroism (CD) analysis, transmission electron microscopy (TEM) and cross-linking examination of both KfrA_R751_ and KfrA_RA3_, were performed to learn about their physico-chemical properties and to decipher the KfrA-type proteins role in plasmid biology, vital for plasmid stability.

We confirmed the high alpha-helical content, oligomerization and the potential to form coiled-coil structures by both studied KfrAs. Structural modeling has revealed that coiled-coil domains (CC domains) formed in KfrAs display local structural deviations, providing mechanistic and functional insights into biological role of the CC domain.

Biomolecular interactions were analyzed by two independent techniques: electrophoretic mobility shift assay (EMSA) and transmission electron microscopy (TEM). Using these techniques, we identified KfrA-type proteins as a novel family of plasmid-encoded proteins, that self-assemble into filamentous structures in the presence of specific DNA sequences. CD spectra showed loss of alpha-helical content of KfrA proteins at elevated, physiological temperatures.

## Results

### Structures of KfrA_R751_ and KfrA_RA3_ exhibit high alpha-helical content

To gain information about the structure and properties of KfrA-type proteins, KfrA_R751_ and KfrA_RA3_, the appropriate ORFs were cloned into pET28M plasmid [28]. The pET28M derivatives, pMAB28.1 T7p-*kfrA*_R751_ and pESB6.59 T7p-*kfrA*_RA3_ were transformed into *E.coli* BL21(DE3) strain (Supplementary Table S.1). The N-terminally 6xHis-tagged proteins were overproduced and purified by affinity chromatography under non-denaturing conditions, as described for KfrA_R751_ [10]. The majority of each overproduced KfrA protein appeared to be soluble and was recovered in the supernatant fraction. For CD 6xHis-tags were removed by thrombin digestion and KfrA proteins were re-purified by FPLC (see Methods).

**Fig. 2:**
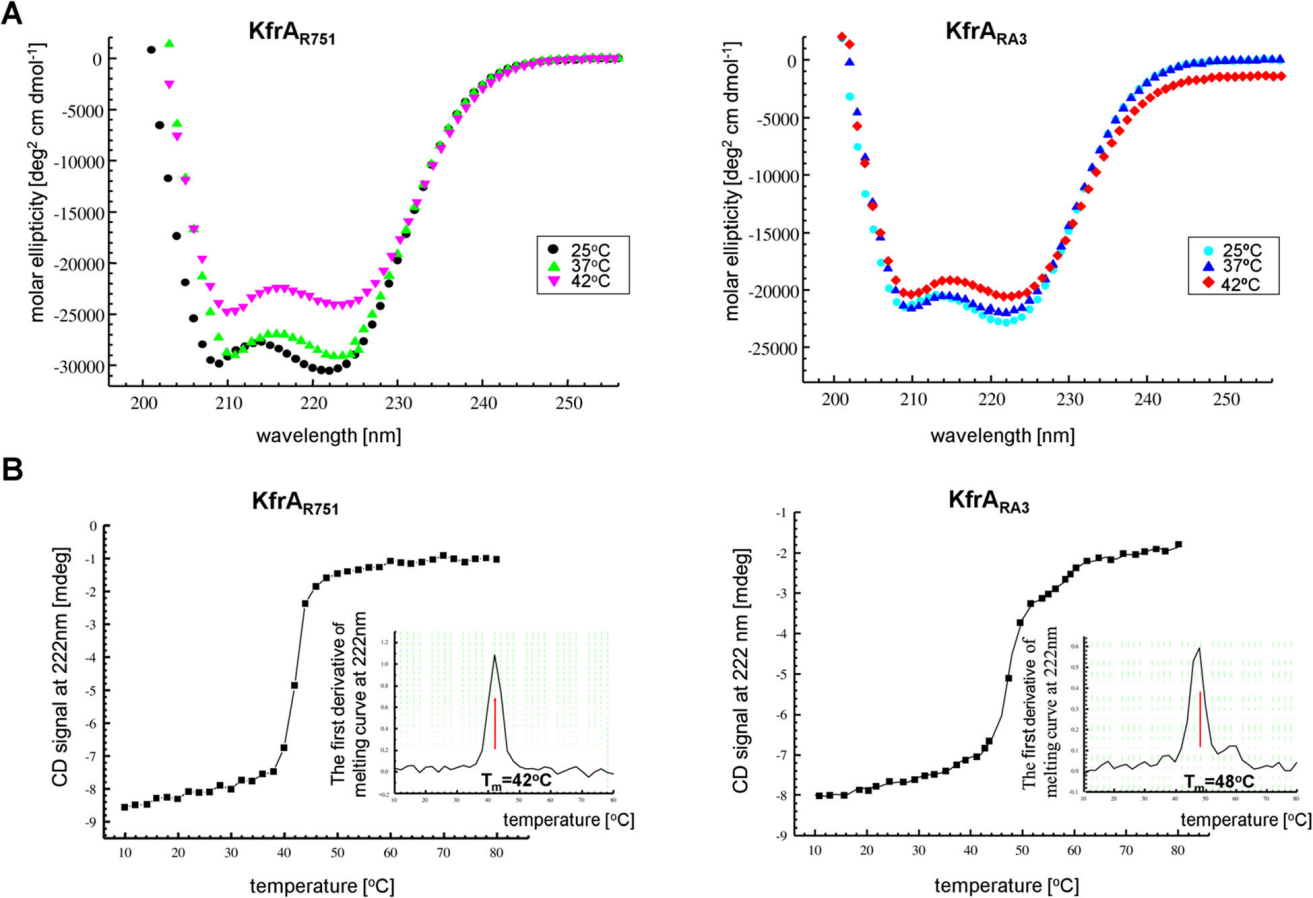
Circular dichroism analysis of KfrA proteins of R751 and RA3 plasmids. KfrA proteins were overproduced and purified by affinity and size-exclusion chromatography. **a** Molar ellipticity was measured at 0.8 μM KfrA_R751_ and 1.4 μM KfrA_RA3_ concentrations at 200–260 nm wavelength range at three temperatures. **b** Melting curves for both KfrAs. The same concentrations of proteins were used as above. The CD signal was measured at 222 nM in the range of temperature 10 - 90 °C with an interval of 2 °C. Inlets correspond to T_m_ (melting temperature) calculations

As indicated in Fig. 2a, at 25 °C KfrA_R751_ and KfrA_RA3_ exhibited distinct CD minima at 208 nm and 222 nm, that are characteristic of alpha-helical proteins and helix-helix interactions. Data deconvolution with CDNN software suggested that the α-helical content of KfrA_R751_ was 86%, with 7% β-strands, and 7% unordered structure (random coil). Slightly less α-helical content (59%) was detected for KfrA_RA3_, with 14% β-strands and 19% of random coil. The results are consistent with previously obtained data for KfrA_RK2,_ homolog encoded by RK2, IncP-1α plasmid [11] and also comply with KfrA_R751_ and KfrA_RA3_ structural predictions (Fig.1a-b) obtained with Quick2D and DeepCoil tools of the MPI Bioinformatics toolkit [29].

It has been observed before that the repression ability of KfrA_R751_ depended on temperature [10]. Therefore, CD spectra of KfrA_R751_ and KfrA_RA3_ were monitored at elevated temperatures. KfrA_R751_ clearly shows structural changes at elevated temperature (42 °C) compared to 25 °C (Fig. 2a). KfrA_RA3_ retained its alpha-helix structure throughout the full range of applied temperatures up to 42 °C (Fig. 2b). The monitoring of proteins’ secondary structures in the temperature range between 10 °C and 80 °C demonstrated that the melting temperature, Tm, for KfrA_RA3_ was higher (48 °C) than for KfrA_R751_ (42 °C), which explained the decrease in KfrA_R751_ alpha-helical content at this temperature (Fig. 2a).

Summarizing, the comparative structural analysis of KfrA proteins confirmed conservation of their secondary structure, which is highly alpha-helical. Nevertheless, thermal stability of the alpha-helical domains of KfrA-type proteins may show different patterns, judging by the example of the two analyzed representatives of this protein family.

### KfrA proteins can self-assemble and exist in multiple oligomeric states in vitro

**Fig. 3:**
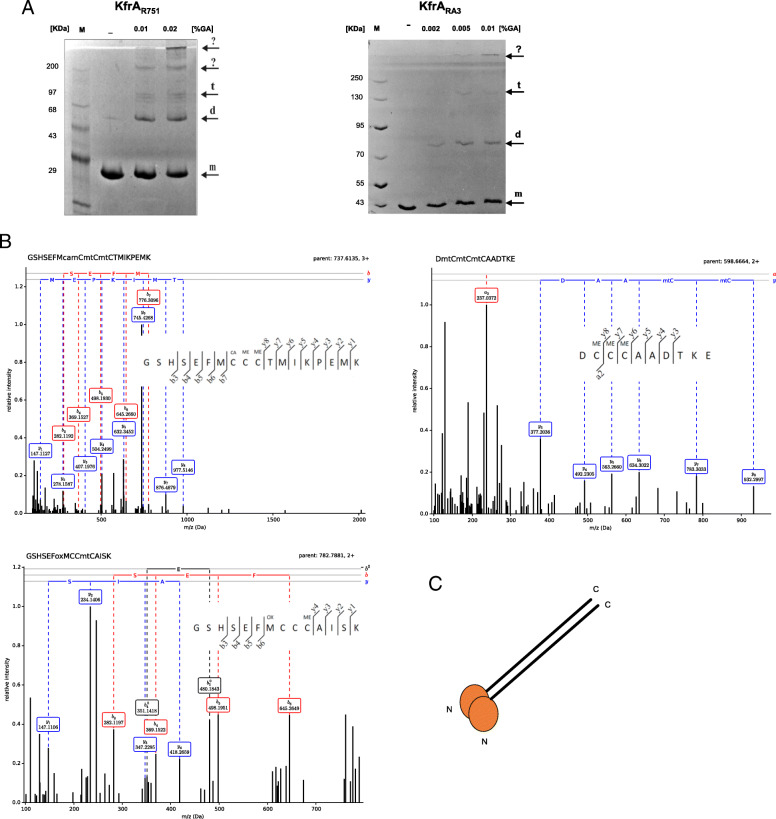
In vitro dimerization of KfrA proteins from R751 and RA3 plasmids in a parallel fashion. **a** The purified His-tagged KfrA proteins (0.1 mg ml^− 1^) were incubated with different concentrations of glutaraldehyde (GA) for 20 min at room temperature and the products were separated on 12% SDS-polyacrylamide gel. Dimers, tetramers and other higher order complexes are indicated by arrows. M- protein markers. **b** Mass spectrometry analysis of KfrA proteins. Free cysteines were covalently blocked with iodoacetamide followed by reduction of disulfide bonds and tagging of the cysteines with methyl methanethiosulfonate. After tryptic digestion of protein, the peptides were subjected to MS/MS analysis. Selected spectra of cysteine-containing peptides identified with highest score are presented. Amino acids preceding ATG codon of KfrAs correspond to N-terminal extension of His-tagged proteins. Modifications resulting from reaction with the cysteine-blocking agents: CAM – Carbamidomethyl; MA – Methylthio are marked. **c** Schematic representation of KfrA homodimer with monomers in parallel arrangement

The in vivo dimerization of KfrA_R751_ has been demonstrated experimentally earlier in the yeast two-hybrid system [10]. AFM measurements at low pH 3.5 showed that, in solution, the major form of KfrA_R751_ protein was tetrameric [27, 30]. We performed additional analysis with purified KfrA_R751_ and KfrA_RA3_ treated with the cross-linking agent (glutaraldehyde) to verify whether the proteins are able to form other multimeric structures and whether experimental conditions affect the oligomeric state of the proteins. The cross-linking reaction was very efficient even at low protein concentration (0.1 mg ml^− 1^) and permitted the visualization of a broad range of KfrA multimers in vitro. At pH 8.5 the significant fractions of both proteins were dimeric (Fig. 3a). Bands corresponding to tetramers and higher multimers with estimated molecular weight (MW) above 200 kDa, were also observed (indicated by arrows). The results clearly demonstrated that, in solution, KfrA proteins may form various higher-order complexes.

The prokaryotic analogues of KfrAs - SMCs, are known to dimerize in an anti-parallel fashion [31, 32]. To establish the anti-parallel or parallel arrangement of monomers in the KfrAs homodimers, the series of KfrA_RA3_ and KfrA_R751_ derivatives were designed with triple cysteine added either within their N-terminal or C-terminal part and the mass spectrometry methods (MS) were used (Materials and Methods).

All modified KfrAs derivatives were over-produced in BL21(DE3)/pET28 system, purified and sequentially blocked with iodoacetamide (IAA) and methyl methanethiosulfonate (MMTS). In the first step, unbound sulfhydryls (−SH) groups of cysteines not involved in dimer formation, if there were any, were alkylated with IAA resulting in their modification to carbamidomethyl (−S-S-CH_2_CONH_2_). After reduction of disulfide bonds, MMTS converted sulfhydryls (−SH) groups resulting in their modification to dithiomethane (−S-S-CH_3_). The chemically modified peptides cleaved with trypsin were subsequentially subjected to LC/MS-MS analysis. We presented the experimental data obtained for three KfrA derivatives named N3Cys-KfrAΔα_R751,_ N3Cys-KfrA_RA3_ and KfrA_RA3_ -C3Cys that, after treatment with trypsin, resulted with efficiently ionized terminal peptides to produce MS/MS data. The N3Cys-KfrAΔα_R751_ derivative represents KfrA_R751_ with internal deletion of 70 amino acids (136Ala-206Ala) [10] within long alpha-helical tail. Prior to measurements by MS, we confirmed, that this derivative can efficiently form dimers in vitro and shows similar polymerization ability as the full length KfrA_R751_ (Supplementary Fig. S.1). As indicated in Fig. 3b, all the cysteines in the C-terminal peptide of KfrA_RA3_-C3Cys were found evenly modified with MMTS, which means that all C-terminal cysteines were bound in a formed dimer, whereas N-terminal 3Cys peptides in KfrA_RA3_ and KfrA_R751_ derivatives produced a partial modification pattern. Detection of the methyl-thio-modified cysteines, regardless of the versions of KfrA proteins used for MS measurements (C-terminally or N-terminally modified) (Fig. 3b), led to the conclusion that C-terminal, as well as N-terminal residues in dimeric KfrA proteins, were proximal to each other. According to MS/MS data, the KfrA_RA3_ and KfrA_R751_ proteins formed parallel dimers (Fig. 3c).

### Structural modeling and sequence analysis

To get some insights into the function of KfrA_RA3_ and KfrA_R751_ proteins, we performed in silico modeling of their coiled-coil domains followed by the molecular dynamics simulations of the top-scored models. For the modeling, we used the “Fold-and-dock” protocol of the Rosetta suite, in which, the input polypeptide chains are simultaneously folded and docked to each other under symmetry constrains ensuring the parallel orientation of helices. Such simulation results in tens of thousands of 3D models ranked according to their estimated folding free energy.

**Fig. 4:**
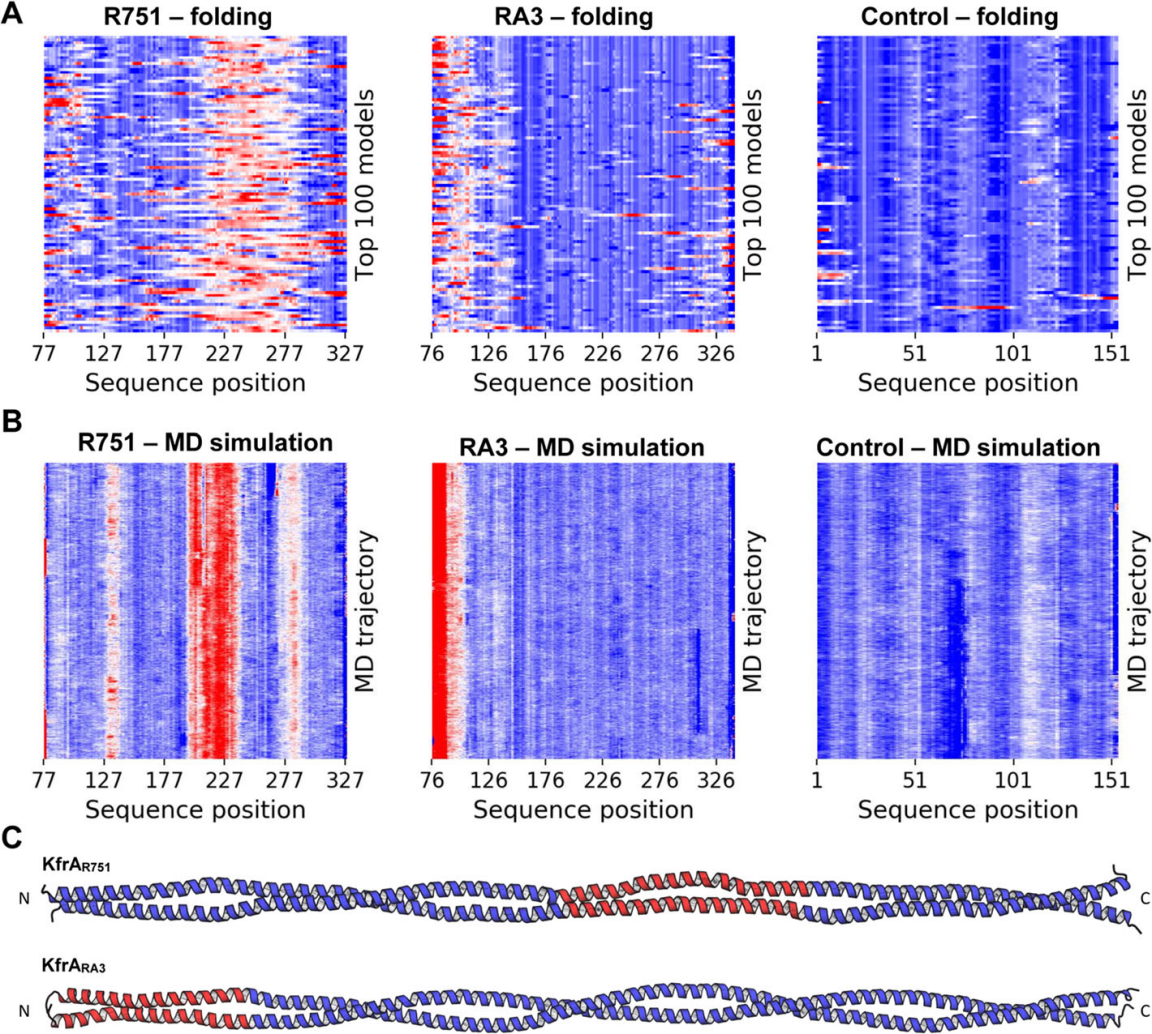
Folding and molecular dynamics simulations of KfrA_R751,_ KfrA_RA3_ and a control coiled-coil domain. **a** Structural parameters of the first 100 best-scored models KfrA_R751_ (residues 77–327) and KfrA_RA3_ (residues 76–326). Each row corresponds to a single model. The best-scored models are located on the top. The local degree of bundle supercoiling is indicated with a color gradient that spans from blue (left-handed) through white (no supercoiling) to red (right-handed). **b** Molecular dynamics trajectory colored as in (**a**). **c** Models extracted from the end of the MD simulations (in Panel **b**). The non-canonical regions are shown in red

Most of the parallel dimeric coiled-coils adopt canonical left-handed structures, whereas other types of arrangements are rare due to reduced stability [8]. Investigation of the top-scored KfrA_R751_ and KfrA_RA3_ CC domain models with a modified version of the SamCC program [33] revealed that both of them contain regions with a high propensity of adopting non-canonical conformations (Fig. 4a). In KfrA_RA3_, this region spans around 50 residues at the N-terminus of the coiled-coil domain, whereas in KfrA_R751_ it is wider and localized centrally. To investigate the dynamics of these local distortions, we performed 100 ns MD simulations of the best-scored R751 and RA3 models (Fig. 4b). All systems converged, by the means of stabilization of the RMSD values (Supplementary Fig. S2), during the first 10 ns of simulation. In the trajectories obtained, the non-canonical regions are constantly present, suggesting that they were not artifacts of the folding procedure and that they are an inherent feature of isolated KfrA coiled-coil domains. As a control, we performed folding and MD simulations for a dimeric coiled-coil of a similar length and a stable canonical left-handed conformation supported by the experimental structure (PDB code: 2efr). The obtained results confirmed, that the used approach is robust and that the presence of non-canonical regions is a characteristic feature of the two analyzed KfrA proteins (Fig. 4c).

**Fig. 5:**
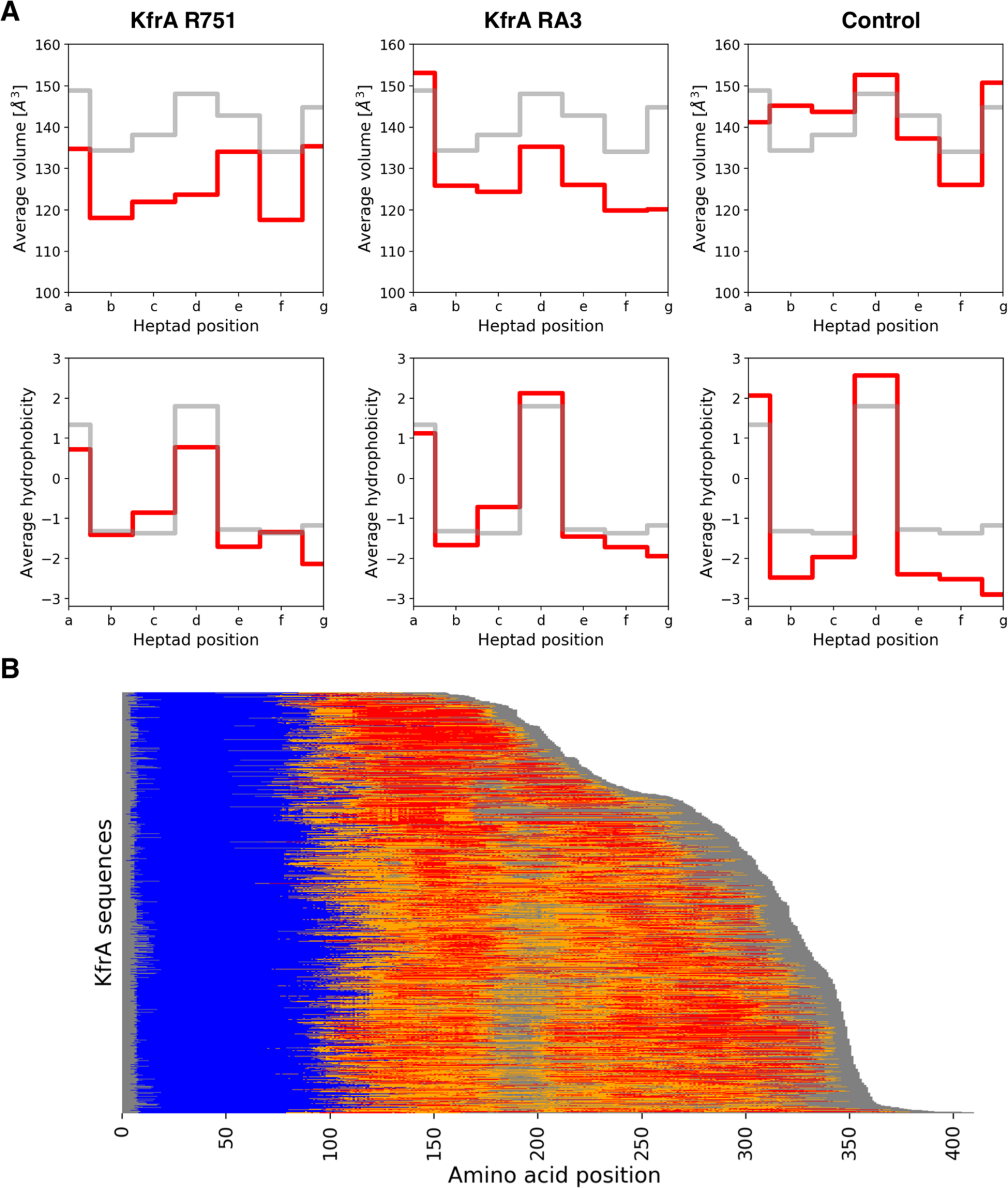
Amino acid composition of KfrA_R751_, KfrA_RA3_ and the domain structure of KfrA homologs. **a** Grey lines indicate average values calculated in a reference set of 800 parallel dimeric coiled-coils (KfrA_R751_ residues 77–327 and KfrA_RA3_ residues 76–326), whereas the red lines depict values obtained from a given sequence. The seven heptad positions group residues according to their position relative to the coiled-coil bundle hydrophobic core (positions a and d are facing the core; b, c, and f the solvent, while e and g are in-between). **b** Each row denotes a single full-length KfrA sequence. Blue regions indicate the presence of HTH domain (PFAM family KfrA_N), whereas orange and red regions correspond to coiled-coil domains predicted with medium (*p* ≥ 0.5) and high (*p* ≥ 0.75) confidence. Grey background indicates the actual length of an individual KfrA homolog

To check whether these structural features are reflected at the sequence level, we compared sequences of KfrA_R751_, KfrA_RA3_, and the control domain to a reference set of 800 parallel dimeric coiled coils sequences obtained from the SamCC-Turbo database (Szczepaniak et al., submitted). To this end, all the residues in each sequence, were grouped according to their positions with respect to the bundle axis and then, for each group, the average hydrophobicity and side-chain volume were calculated (Fig. 5a). In terms of hydrophobicity, R751, RA3 and the control domain displayed properties similar to those seen in the reference set. However, both KfrA CC domains deviated substantially from the background in terms of the sidechain size. Detailed analyses (Supplementary Fig. S3) revealed that they contain considerably more alanine and other small residues, a feature associated with the decreased stability of CC domains [34]. This observation indicates that KfrA CC domains not only display local structural deviations but are also destabilized by the atypical amino acid composition.

Finally, we checked whether KfrA_R751_ and KfrA_RA3_ are prototypical members of the KfrA family. To this end, using BLAST we obtained a non-redundant (max. 90% sequence similarity) set of 1020 sequences containing regions homologous to the HTH domains of these two proteins and analyzed them with DeepCoil [35] (Fig. 5b). In 97% of them, coiled-coil domains were predicted C-terminally to the HTH domain with high confidence (probability of 0.75 or more), confirming that the domain architectures observed in KfrA_R751_ and KfrA_RA3_ proteins, i.e. HTH domain followed by a CC domain, are representative for the whole family. Intriguingly, we found that 84% of these CC domains contain apparent deviations from the canonical heptad pattern. This is in striking contrast to the reference set of parallel dimeric coiled coils sequences among which only 20% contained non-canonical segments. Such a difference further supports the notion that the presence of non-canonical regions is specific for the KfrA family and, as discussed above, may be essential for their function.

### KfrA proteins of RA3 and R751 specifically bind their OK motifs in vitro

**Fig. 6:**
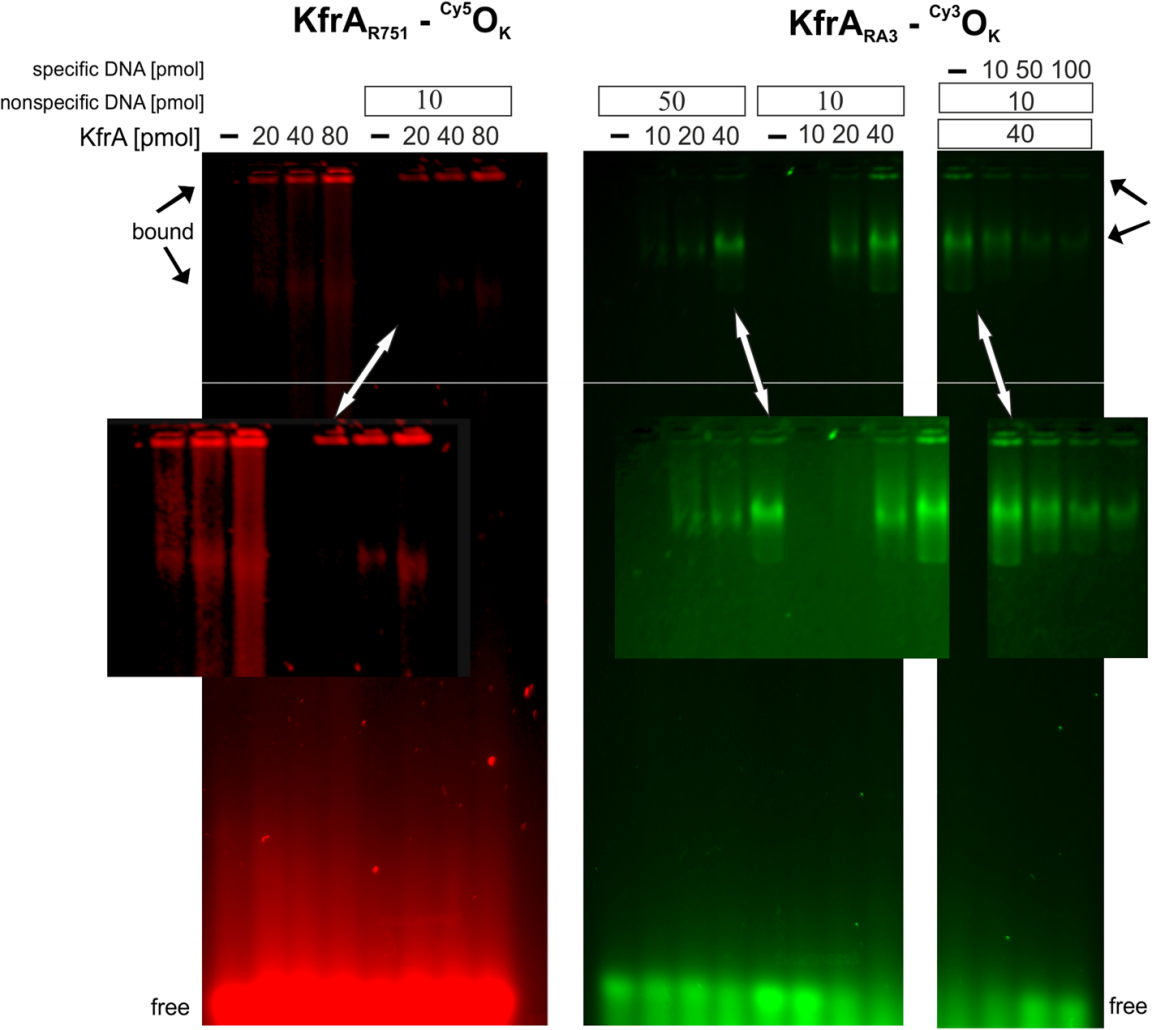
DNA binding activity of KfrA proteins from R751 and RA3 plasmids assayed by EMSA. Appropriate His-tagged KfrA proteins were incubated for 30 min at 25 °C with 10 pmols of fluorescently labelled ds oligonucleotides corresponding to OK_R751_ (oligonucleotides #15/#16 labelled with Cy5) or OK_RA3_ (#17/#18 labelled with Cy3). Double stranded oligonucleotides (annealed #5 and #6) were used as nonspecific DNA in binding reactions separated on 2.5% agarose gels run in 0.5xTBE. The right panel for KfrA_RA3_ demonstrates the results when the increasing quantities of unlabeled competitive ds oligonucleotide (annealed #9 and #10 oligonucleotides) was added to the binding reactions

The role of KfrAs as the transcriptional regulators of the cognate promoters has been previously demonstrated for KfrA_RK2_ [11] and KfrA_R751_ [10] and postulated for KfrA_RA3_ [26]. To confirm the localization of their binding sites, the purified KfrA_R751_ and KfrA_RA3_ were used in the Electrophoretic Mobility Shift Assay (EMSA) with Cy3-, or Cy5-labelled DNA fragments, respectively. His_6_-tagged KfrA_R751_ bound specifically to the 50 bp DNA fragment containing putative KfrA operator OK_R751_ (highlighted in green (Fig. 1c), although, the complex formed was unstable and dissociated as indicated by the “smear” on the gel (Fig. 6). At a range of KfrA_R751_ concentrations, used in the assay, two main complexes were always observed (as indicated with black arrows Fig. 6), one with a very high molecular weight (MW), which prevented the protein-DNA complex from entering the agarose matrix and a second complex of lower MW. Much higher specificity of binding was demonstrated by KfrA_RA3_ towards 60 bp DNA fragment containing OK_RA3_ motif, although “smearing” was also observed (Fig. 6 middle panel). Two major forms of KfrA_RA3_–DNA complexes were detected and are indicated by black arrows (Fig. 6). According to the data published in our previous report [10], the purified KfrA_R751_ demonstrated a range of stable SDS resistant multimers, that were visualized by Western blot analysis with antibodies against KfrA. Judging on the previous and present data (Fig. 3a-b), it can be concluded that KfrA proteins may bind to their operator in multiple oligomeric forms. The specificity of KfrAs DNA binding was confirmed in the competition experiments. The presence of unspecific (unlabeled) oligonucleotides, slightly decreased DNA binding affinity of KfrA_R751_ (left panel Fig. 6) and KfrA_RA3_ (middle panel Fig. 6) as expected for DNA binding protein. The specific unlabeled 60 bp fragment with OK_RA3_ successfully outcompeted the labeled fragment when added at increased concentrations to the binding reactions with Cy5 labeled oligonucleotide (right panel Fig. 6). In conclusion, the specificity of the KfrA_R751_- and KfrA_RA3_-DNA interactions was confirmed and DNA sequences of OK_R751_ and OK_RA3_ operators verified.

### KfrA proteins assemble into elongated filamentous structures when bound to the O_K_ operator motif

**Fig. 7:**
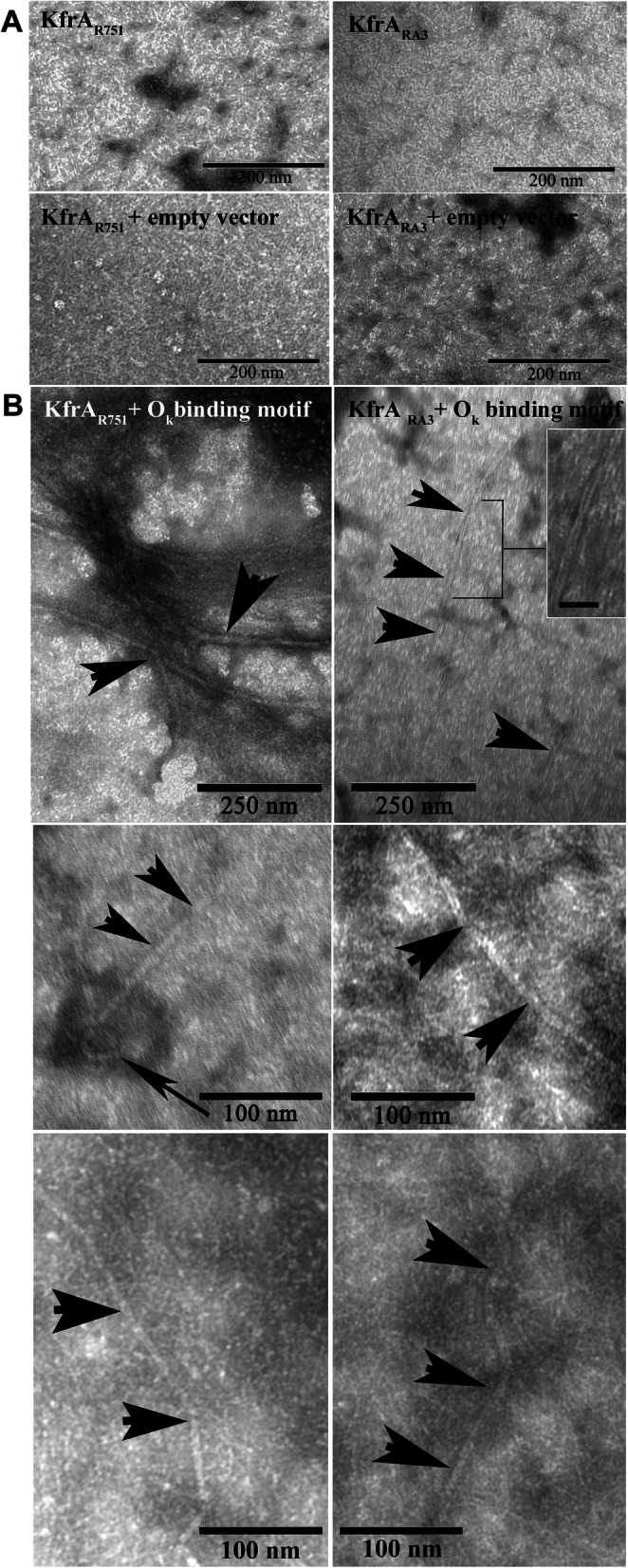
Images of KfrA proteins in the presence of specific DNA binding sites. **a** KfrA proteins at concentration 0.3 mg ml^− 1^ in 50 mM sodium phosphate pH 7.5, 150 mM NaCl buffer were visualized by transmission electron microscopy (TEM) without plasmid DNA or with pUC18 DNA as the control (12.5 μg ml^− 1^). **b** Filaments of KfrA_R751_ and KfrA_RA3_ were formed (as indicated by black arrows) after incubation with pUC18 derivatives, respectively pMAB18.7/8 and pESB2.68, carrying native, specific OK motifs. Inset shows magnified view of the braided fibrillar structure, the scale bar - 50 nm

By using in vitro approach, we confirmed that KfrA_R751_ and KfrA_RA3_ could bind to OK_R751_ and OK_RA3_ motifs, respectively. The next question was whether the KfrAs-DNA binding affected the proteins’ structure. To visualize KfrA proteins in the presence or absence of DNA, TEM was applied. First, both proteins with no DNA in solution were examined. The observation of KfrA_R751_ by TEM correlated with previous studies conducted by AFM, which visualized protein complexes of elongated, rod-like shape [27]. Similar protofilaments were also visualized for KfrA_RA3_ (Fig. 7a upper row). The presence of control plasmid DNA, pUC18 without cognate OK binding motifs, in either KfrA_R751_ or KfrA_RA3_ protein solutions facilitate some detectable structural changes in organization of protofilaments, which suggest non-specific interaction with control plasmid DNA (Fig. 7a, the second row).

The TEM data of KfrA_R751_ and KfrA_RA3_ complexes with plasmidic DNA carrying OK_R751_ and OK_RA3_ motifs, respectively, revealed a range of structures such as polymers of different width and length, that exceeded 500 nm in length (indicated by arrow heads Fig. 7b), “hawser-like” supercoiled filaments (indicated by inset Fig. 7b, upper row at the right) and ring-like structures, which were not present after incubation with pUC18 (empty vector). The in vitro data obtained by TEM*,* suggest that binding to OK -motifs on DNA can trigger self-assembly of shorter KfrA proto filaments into the higher order, coiled-coil, mainly rod-like structures.

## Discussion

During cell division, the low-copy-number plasmids are precisely distributed to each daughter cell, due to NTPases driven spatiotemporal directional relocation across the host nucleoid. There are two systems, providing plasmids an efficient active partitioning, either based on an actin-like ATPase (Type II) activity or dependent on tubulin-like GTPas (Type III) motor proteins, that execute unidirectional relocation of DNA along the long axis of the cell/nucleoid preventing transverse plasmid motion [5, 36]. The most frequently occurring Type I active partitioning system relay on the function of ParA Walker-type ATPase, that oscillates between the cell poles is still far from being understood [37]. The fundamental mechanism linking ParA dynamics with regular plasmid positioning is still unclear.

KfrAs proteins with N-terminal DNA-binding domain and C-terminal, an alpha-helical, SMC-like domain (Structural Maintenance of Chromosomes) have been annotated for numerous broad-host-range plasmids of IncP, IncU, IncW, IncA/C and PromA groups, clinical isolates of human origin and isolates from agriculture soils [22, 24, 38–46]. KfrA proteins, were postulated to enhance the stability of broad-host-range plasmids with Type I active partition systems [10, 11]. Our report addresses the important biological issue, how the coiled-coil KfrA-type proteins may assist plasmid DNA segregation. They may assemble into a scaffold of alpha-helical filaments or act as SMC-like proteins in condensation of plasmid DNA for transport and proper positioning in a cell. KfrA proteins, due to their structure, are challenging to study. We used complementary analytical approaches, both sensitive and qualitative, to study in parallel KfrA_R751_ and KfrA_RA3_. Better understanding of biophysical properties of KfrA-type proteins shed light on understanding the biological role of this class of proteins in bacterial host cells.

We confirmed by CD (Fig. 2), EMSA (Fig. 6) and TEM (Fig. 7) that both KfrAs displayed very similar structural and functional properties, despite their low amino acid sequence conservation. They are plasmid-encoded proteins of high alpha-helical content, possess DNA binding activity towards cognate OK motifs (Fig. 6) and, due to the interaction with DNA carrying OK motifs, can polymerize into double-helical filaments in vitro in an ATP-independent and DNA-dependent manner (Fig. 7 Panel b). As a novel class of plasmid-encoded filamentous proteins, the double-helical filaments of KfrAs share morphological similarity with cytomotile/cytoskeletal proteins, such as CdvA, a component of CdvABC ESCRT III like complex, involved in the cell division of hyperthermophilic archaeon *Metallospherae sedula* [47], and ParA2, chromosome segregation protein of *Vibrio cholerae* [48]. These proteins also form polymers in a DNA-dependent manner. However, in contrary to CdvA and ParA2, which do not contain any conventional DNA binding domain, plasmid-encoded KfrA_R751_ and KfrA_RA3_ proteins possess HTH domain [49], to recognize specific motifs in plasmid DNA. The OK_R751_ and OK_RA3_ binding sites (Fig. 1c-d), which we confirmed as the highly specific binding sites for the KfrA_R751_ and KfrA_RA3_, respectively, are required for cognate proteins self-assembly into polymers (Fig. 7b). There is also a degree of structural similarity between KfrA-type proteins and proteins that contain extensive, segmented coiled-coil domain, such as the cytoskeletal intermediate filament-like IF proteins, which function in cell shaping [2].

KfrAs share a few structural features with SMC proteins. Firstly, they form coiled-coil domains, secondly, these domains are destabilised by short regions of non-canonical helical motifs (Fig. 4). Regardless of sharing structural features with the prokaryotic SMC proteins, we doubt that the biological significance of KfrA proteins relies on the condensation or cohesion of plasmid molecules. A typical, evolutionary conserved SMC protein exhibits Walker-type ATPase activity with characteristic nucleotide-binding motifs “Walker A” in the N-terminus and “Walker B” motif in C-terminus, that are not present in KfrAs reported to date. Furthermore, KfrAs differ from SMCs by parallel arrangement of monomers in the dimeric forms, which we showed during this study by MS data (Fig. 3). Lack of indication of KfrA anti-parallel dimers formation makes the alternative hypothesis more plausible. KfrA filaments might assemble into a scaffold for plasmids DNA transport during the DNA partitioning process. In this scenario, interaction of KfrA-type proteins with the plasmid encoding partitioning proteins ParA/IncC and ParB/KorB, in Type I partitioning system [50, 51] would be required, to form the most complex segrosome discovered to date. In parallel studies, it was shown that KfrA of RA3 indeed interacts with the segrosome proteins, KorB and IncC [52]. Such complex may assemble for plasmid DNA transport and mechanical energy from the thermal Brownian motion, may provide driving force, as postulated by T. Yanagida *et. al.* [53]. Active movement by molecular motors, which is distinguished from diffusion movement is directed in one direction. Directional movement requires energy, that is released from breakdown of ATP to ADP [54] and that might be delivered by the segrosome component IncC.

Notably, KfrA-type proteins, in addition to their confirmed biological role as DNA binding transcription repressors, may also act as intracellular temperature sensors. We provide evidence, that temperature in the physiological range between 37 °C and 42 °C contributes to structural changes in KfrA_R751_ through partial unfolding of the alpha-helical domain in vitro (Fig. 2). This observation makes the temperature sensitive, alpha-helical domain in KfrAs, a thermo sensing domain. Previously, it has been shown for the homologue of KfrA_R751,_ alpha-helical TlpA protein of the pLT2 plasmid of *S. typhimurium*, that its specific DNA binding activity is also strongly temperature dependent [55]. TlpA function has not yet been elucidated, although it was suggested that it might act as a virulence factor/temperature sensor at the entry of pathogenic bacteria into an eukaryotic host such as chicken [56].

It is tempting to draw an analogy between CC domains of KfrA and *Streptococcus* M1 virulence factor [57, 58]. It has been shown that the presence of destabilizing residues and non-canonical conformations promotes conformational dynamics which, in turn, is necessary for the interaction between M1 and its partner, fibrinogen. We hypothesize, that the intrinsic instability of KfrA CC domains (Fig. 4 and Fig. 5) may have a similar function and facilitate the formation of alternative conformational states, one of which is capable of the filament formation. Such an aggregation-promoting conformation may become dominant upon DNA binding to the HTH domain. We envision that this hypothesis could be validated by designing KfrA variants lacking the non-canonical regions in their coiled-coil domains and testing their ability for filament formation and interaction with partner proteins.

KfrA family contains over 1000 members with typical domain architecture, where HTH domain is followed by a CC domain of different length (Fig. 5b). From the perspective of plasmids inheritance, the mechanism underlying intracellular dynamics of KfrA filaments formation during bacterial cell division, as well as full understanding of which protein complexes associated with KfrA assist plasmid delivery to the ¼ -¾ positions for segregation, may display more complexity than presently envisioned. Recent in vitro reconstitution experiments and in vivo super-resolution microscopy have provided strong evidence against a ParA filament-based plasmid movement mechanism for Type I ParA/B systems [59–62]. Dynamic ParA cloud moving along the nucleoid is sufficient for plasmid segregation, but alternatively ParAs might also act via a cytomotive filamentation mechanism in the complex with KfrAs, as accessory proteins to the Type I partition system in certain systems. Therefore, one of our future efforts will be to provide evidence of formation of KfrA polymeric structures in the complex with partition proteins at physiological concentration in vivo.

## Methods

### Bacterial strains and growth conditions

*Escherichia coli* strains used were K12 strain DH5α F^*−*^(Φ*80ΔlacZM15*) *recA1 endA1 gyrA96 thi-1 hsdR17*(r_k_^−^m_k_^+^) *supE44 relA1 deoR (lacZYA-argF)U169*, *E. coli* B strain BL21 F^−^ *ompT hsdS*_B_ (r_B_^−^m_B_^−^) *gal dcm* (λDE3) (Novagen Inc). Bacteria were generally grown in L broth or L agar (L broth with 1.5% w/v agar) at 37 °C supplemented with antibiotics as appropriate: benzyl penicillin, sodium salt (150 μg ml^− 1^ in liquid media and 300 μg ml^− 1^ in solid media) for penicillin resistance*,* kanamycin sulphate (50 μg ml^− 1^) for kanamycin resistance, chloramphenicol (10 μg ml^− 1^) for chloramphenicol resistance or tetracycline (10 μg ml^− 1^) for tetracycline resistance. The L agar used for blue/white colony screening contained 0.1 mM IPTG (isopropyl-β-D- thiogalactopyranoside) and X-gal (5-bromo-4-chloro- 3-indyl-β-D-galactoside) at 40 μg ml^− 1^.

### Plasmids

The plasmids used in this study are listed in Supplementary Table S1. Plasmids for KfrAs purification were constructed by inserting the *kfrA*_R751_ and *kfrA*_RA3_ open reading frames into pET28m, a derivative of pET28a Km^R^ [10, 63] (Novagen Inc) modified with the help of the synthetic oligomer so that a His_6_-tag and thrombin cleavage site precede almost directly the *EcoRI* site [28]. Purified His-tagged KfrAs and their derivatives were N-terminally extended by the sequence MGSSH_6_SSGLVPRGSHSEF.

### Plasmid DNA isolation, analysis, cloning and manipulation of DNA

Plasmid DNA was isolated by standard procedures [64]. Digestion of plasmid DNA with restriction enzymes was carried out under conditions recommended by suppliers and run on agarose gels of concentration 0.8 to 2.0% (w/v). Standard PCR reactions were performed as described previously [10] with the pairs of primers listed in Supplementary Table S2. Oligonucleotides carrying KfrAs binding motifs were mixed at concentration of 100 μM, incubated in a heat block at 95 °C for 5 min and annealed in a cooling heat block until the temperature reached 25 °C.

Synthesis of oligonucleotides was performed by Genomed and Future Synthesis. DNA sequencing by Genomed and IBB, PAS, Warsaw sequencing core facility using Dye terminator kits supplied by the manufacturer. Sequences were compared to Genbank/EMBL databases.

### Purification of His_6_-tagged KfrA proteins

Exponentially growing BL21(DE3) transformed with pMAB28.1 or pESB6.59, were induced as described previously [10] with 0.5 mM IPTG at a cell density of approximately 2 × 10^8^ cfu ml^− 1^ and grown for an additional 2–3 h with shaking at 37 °C. The cells from 200 ml cultures were harvested by centrifugation, sonicated in a sonication buffer 50 mM sodium phosphate buffer pH 7.5, 300 mM NaCl supplemented with 1 mg ml^− 1^ lysozyme, Protease Inhibitor Cocktail (Sigma-Aldrich) and centrifuged at 28000 rpm at 4 °C to obtain a cleared supernatant. The supernatant was loaded on ProtinoTED 2000 packed column (Macherey-Nagel) pre-equilibrated with the sonication buffer. Bound His_6_-tagged proteins were washed with 50 mM sodium phosphate buffer pH 7.5, 300 mM NaCl and then eluted with elution buffer 50 mM sodium phosphate pH 7.5, 300 mM NaCl with 250 mM of imidazole [65]. Purified proteins were buffer exchanged using 5 ml HiTrap Desalting Columns (GE Heathcare) into DESALT buffer 50 mM sodium phosphate pH 7.5, 300 mM NaCl. To ensure batch-to batch consistency the purification procedure was monitored by SDS-PAGE using Phast gel system (Pharmacia) and UV spectroscopy between 240 nm and 340 nm. The proteins folding and stability in time was confirmed using nanoDSF (Prometheus NT.48 NanoTemper). Proteins were used freshly or stored for a week at 4 °C prior for downstream procedures and measurements.

### Glutaraldehyde cross-linking

KfrAs at concentration 0.1 mg ml^− 1^ were incubated at 25 °C with different concentrations of glutaraldehyde in 0.05 M bicine-NaOH buffer (pH 8.5), 0.1 mM DTT, 0.4 M NaCl for 20 min and the reaction was quenched by adding ethanolamine-HCl (pH 8) to a final concentration of 0.14 M. The products were analyzed by SDS-PAGE using precast 12.5% polyacrylamide gels and Phast gel system (Pharmacia).

### Protein identification by mass spectrometry

KfrA derivatives containing three cysteines either on N-terminal or C-terminal end were overproduced from plasmids pSRA28.1, pSRA28.2, pSRA28.3 (Table S1) and purified by IMAC affinity chromatography. The protein solutions were further treated according to FASP method with major modifications to allow sequential blocking of cysteines. Unbound cysteines were alkylated with 30 mM iodoacetamide in 100 mM ammonium bicarbonate pH 7.4 and protected from light, for 35 min at room temperature. The solutions were transferred onto Vivaspin 500 filter with 30 kDa cutoff, (Sartorius) and washed twice with 8 M urea in ammonium bicarbonate buffer (14,000 g, 30 min, 20 °C). Disulfide bonds were reduced with 60 mM DTT in urea solution for 40 min at room temperature and washed once with urea to remove the excess of the reagent. Free cysteines were blocked with 80 mM methyl methanethiosulfonate (MMTS) in 8 M urea for 10 min at room temperature and proteins were washed three times with 100 mM ammonium bicarbonate buffer pH 7.4. Proteins were digested overnight at 37 °C with trypsin/LysC mixture, in approximately 1:25 ratio of protease to protein. Peptides were eluted from FASP filter by subsequent centrifugation with 100 mM ammonium bicarbonate and 0.5 M sodium chloride. The resulting peptide mixtures were concentrated and desalted on a RP-C18 pre-column (Waters, Milford, MA), and further peptide separation was performed on a nano-Ultra Performance Liquid Chromatography (UPLC) RP-C18 column (Waters, BEH130 C18 column, 75 μm i.d., 250 mm long) using a 160 min linear acetonitrile gradient in the presence of 0.1% FA. Column outlet was directly coupled to the ion source of the Orbitrap Elite mass spectrometer (Thermo Electron Corp., San Jose, CA), working in the regime of data dependent MS to MS/MS switch. The proteins were sequenced with good sequence coverage ranging from 62 to 83%. MS measurements and data analysis were performed at the Mass Spectrometry Laboratory, IBB PAS.

### Analysis of mass spectrometry data

The acquired MS/MS data were pre-processed with Mascot Distiller software (v. 2.6, MatrixScience, London, UK) and a search was performed with the Mascot Search Engine (MatrixScience, London, UK, Mascot Server 2.5) against the user database, which contain modified sequence of KfrA proteins. To reduce mass errors, the peptide and fragment mass tolerance settings were established after a measured mass recalibration (resulting in values of 5 ppm for parent and 0.01 Da for fragment ions. Further criteria for parameters search were as follows: enzyme, Trypsin; missed cleavages, 1; variable modifications: Carbamidomethyl (C), Methylthiol (C), Oxidation (M); instrument, HCD. The expected value threshold of 0.05, to avoid a random match, was used for the analysis.

### Circular Dichroism (CD) analysis of KfrA proteins

His-tags, from His-tagged KfrA proteins, were removed by incubation with biotinylated thrombin (Thrombin cleavage Capture Kit, Novagen) and the second affinity chromatography procedure followed by size-exclusion chromatography. Size exclusion chromatography on Superdex 200 10/300 GL column (FPLC) equilibrated with buffer 50 mM sodium phosphate pH 7.5, 100 mM NaCl was applied to re-purify KfrA proteins prior to CD analysis [66]. Purification was done at the Laboratory of RNA Biology and Functional Genomics, IBB PAS. Protein concentration was determined using a Carry3E UV-visible spectrophotometer and by amino acid analysis [67]. CD measurements were carried out at Jasco J-815 CD spectrometer, in 1 mm path length quartz cuvettes. The CD spectra were collected twice with an average time 2 s for each point and a step size of 1 nm from 200 to 270 nm. All spectra were adjusted to the buffer. The molar residue ellipticity in (deg cm2 dmol-1) was calculated according to [68]. The CD melting experiments were recorded with the sample heating from 10 to 90 °C, with 2 °C increment, 30 s accumulation time per data point, and 150 s equilibrium time at each temperature. The melting temperature, T_m_ was determined from the first derivative of the protein melting profile. ORIGIN software was used for the data analysis and display.

### Analysis of protein-DNA interactions by electrophoretic mobility shift assay (EMSA)

Complementary oligonucleotides corresponding to KfrAs binding sites (O_K_) labeled at 5′ ends with Cy3 (oligonucleotides #17/#18 for RA3 O_K_) or Cy5 (oligonucleotides #15/#16 for R751 O_K_) were mixed in equimolar quantities. Mixtures were incubated at 95 °C for 15 min and then heat block holder was removed from the heating system and left at a room temperature for cooling and annealing of both strands. Recombinant His_6_-KfrA_R751_ and His_6_-KfrA_RA3_ were incubated with 10 pmols of appropriate specific ds labeled oligonucleotides in KfrAs DNA binding buffer of 50 mM Tris-HCl pH 8.0, 10 mM MgCl_2_ and 50 mM NaCl. Unlabeled ds oligonucleotides #5/#6 were used as unspecific DNA in binding reactions whereas ds nucleotides #9/#10 were used as specific competitor DNA. Reactions were incubated at 37 °C for 30 min and complexes analyzed on 2.5% agarose gels run in 0.5xTBE buffer (90 mM Tris-borate, 2 mM EDTA). DNA was visualized using FluorChemQ MultiImageIII ChemiImager and the images were captured using Alpha View software (Alpha Innotech).

### Transmission electron microscopy (TEM) of purified KfrA proteins and KfrA-DNA complexes

10 μl samples (0.3 mg ml^− 1^ protein in 50 mM sodium phosphate pH 7.5, 150 mM NaCl buffer, without or with 12.5 μg ml^− 1^ plasmid DNA, control pUC18 or plasmids pMAB18.7/8 or pESB2.68) were placed for 40 s on copper grids (400 mesh, Cu, Carbon type B, Ted Pella) and incubated at 25 °C. The grids were negatively stained with 2% (w/v) uranyl acetate (SPI Supplies) for 25 s. The grids were examined in a High-Performance Biology Transmission electron microscope JEM 1400 (JEOL Co., Japan, 2008) equipped with 11 Megapixel TEM Camera MORADA G2 (EMSIS GmbH, Germany).

### Bioinformatics

Boundaries of coiled-coil domains were predicted with DeepCoil [35] and DeepCoil 2.0 (https://github.com/labstructbioinf/DeepCoil/tree/develop; manuscript in preparation) and their oligomerization state was verified with the LOGICOIL [69]. To identify CC domains containing non-canonical segments (in the KfrA family and the reference set of dimeric, parallel bundles), their predicted heptad positions were compared to the pattern expected for the canonical left-handed structures. Sequences containing any discontinuities in the heptad pattern were marked as non-canonical. 3D structures of the KfrA coiled-coil domains were modeled with the “Fold-and-dock” method [70]. Briefly, the algorithm rebuilds coordinates of the symmetric homoligomeric proteins by assembling 3- and 9- amino acid fragments. Due to the size of the investigated domains, the sampled conformational space was constrained by a) selecting amino acid fragments utilized by the “Fold-and-dock” algorithm only from the set of parallel, dimeric coiled-coil domains available in the Protein Data Bank with the use of structure-set fragment picker [71] and b) limiting the coiled-coil orientation by restraining distances between the Cα atoms of the first and last residues of each helix with the flat harmonic potential (AtomPair CA X CA Y FLAT_HARMONIC 10 5 5, where X and Y correspond to the residue numbers). To verify the applicability of the described protocol to the large protein assemblies, a control folding run was performed with a coiled-coil protein of known structure and similar size – the C-terminal fragment of the tropomyosin domain (PDB: 2efr). The resulting folding profile (the Rosetta energy score of the models rebuilt from the sequence only vs the RMSD to the native PDB structure), indicating the validity of the procedure is shown in Supplementary Fig. S.3. To confirm the stability of the three CC domains **(**R751, RA3, and control), we performed explicit solvent MD simulations of their best-scoring models (see Supplementary Materials for details).

## Supplementary Information

**Additional file 1.**

## Data Availability

The datasets used and/or analysed during the current study are available from the corresponding author on reasonable request. **Accession numbers** GenBank: AAC64418.1 KfrA_R751_, GenBank: ABD64835.1: KfrA_RA3_.

## Abbreviations

HTH: Helix-turn-helix
CC domain: Coiled-coil domain
IAA: Iodoacetamide
MMTS: Methanethiosulfonate
