# Supplementary material for: Revealing biophysical properties of KfrA-type proteins as a novel class of cytoskeletal, coiled-coil plasmid-encoded proteins
Source: BMC Microbiol. 2021 Jan 22;21:32. doi: 10.1186/s12866-020-02079-w (PMC7821693; doi:10.1186/s12866-020-02079-w)
Supplement: Supplementary file 1 — Additional file 1. [file 12866_2020_2079_MOESM1_ESM.doc]

Supplementary Material for

***Revealing biophysical properties of KfrA-type proteins as a novel class of cytoskeletal, coiled-coil plasmid encoded proteins***

Adamczyk M^1^*, Lewicka E^2^, Szatkowska R^1^, Nieznanska H^3^, Ludwiczak J^4,5^, Jasiński M^4^, Dunin-Horkawicz S^4^, Sitkiewicz E^2^, Świderska B^2^, Goch G^2^, Jagura-Burdzy G^2^

^1^ Warsaw University of Technology, Faculty of Chemistry, Chair of Drug and Cosmetics Biotechnology, Noakowskiego 3, 00-664 Warsaw, Poland

^2^Institute of Biochemistry and Biophysics PAS, Department of Microbial Biochemistry, Pawinskiego 5a, 02-106 Warsaw, Poland

^3^ Nencki Institute of Experimental Biology PAS, Laboratory of Electron Microscopy, Pasteura 3, 02-093, Warsaw, Poland

^4^ University of Warsaw, Centre of New Technologies, Laboratory of Structural Bioinformatics, , 02-097 Warsaw, Poland

^5^ Nencki Institute of Experimental Biology, Laboratory of Bioinformatics, Pasteura 3, 02-093 Warsaw, Poland.

*Corresponding author’s e-mail address: [madamczyk@ch.pw.edu.pl](mailto:madamczyk@ch.pw.edu.pl)

**Molecular dynamics simulations**

The structures were immersed in the rectangular water boxes, with the longest helical axis centered along the Z-axis of the box. The size of the boxes ensured at least 15 Å layer of TIP3P (1) water molecules in the XY plane around the systems. In the Z-axis, the solvent layer was enlarged to 40 Å in each direction, to allow for the changes of box size during the NPT equilibration. The systems were neutralized via the addition of Na+ counterions, that were placed by replacing water molecules, in the local minima of Coulombic potential.

The initial solvated systems were subjected to energy minimization, 200 steps with the use of the L-BFGS (2,3) algorithm. The systems were then gradually heated from 30 to 300 K in 10 K steps, each step lasting 20 ps. During heating, positional restraints were applied to solute heavy atoms with a force constant of 50 kcal/mol/Å^2. The restraints were then removed over eight 500 ps long rounds, with a force constant reduced in half compared to the previous round. After that, a 5 ns equilibration in the NPT ensemble was performed to adjust the box size with a target pressure set to 1 bar. Following the heating and equilibration, all systems were subjected to the production phase in the NVT ensemble for 100 ns in 300K. Trajectories prior to the production phase were not considered in further analysis.

All simulations were performed with the use of the Amber ff14SB (4) force field. A cutoff of 10 Å to the Lennard−Jones and short-range electrostatic interactions with a switching function becoming effective at 9 Å was applied. Long-range electrostatic interactions were calculated using the particle mesh Ewald (PME) summation method (5) under periodic boundary conditions. SHAKE (6) was applied to allow for a 2 fs time step. The temperature was maintained via a Langevin thermostat with a friction constant set to 5 ps^-1. For the preparation of initial coordinates and topology files, the AmberTools18 (7) was used. The simulations were performed using the OpenMM (8) (version 7.4.1).

During simulations the solute molecules were enclosed in the cylindrical container. The additional harmonic potential was added to the system, with the force constant set to 25 kcal/mol/Å^2 outside the cylinder and to 0 kcal/mol/Å^2 inside. The radius of the cylinder, centered along the Z-axis, was set to 25 Å. The additional force in the system allowed to avoid global changes of the solute structure (such as bending or rotations) that could lead to the interaction of the solute with itself through the periodic boundary conditions.

**Table S.1 List of plasmids used in this work**

| Designation | Description | Source |
| --- | --- | --- |
| pAKB2.60 | pGBT30 *tac*p-*kfrA*_RA3_; PCR amplified *kfrA*_RA3_ with primers #3 and #4 and cloned as an EcoRI-SalI fragment into pGBT30 | ZBD IBB collection |
| pESB2.61 | annealed oligonucleotides #5 and #6 cloned between EcoRI and SalI sites in pUC18 | This work |
| pESB2.68 | RA3O_K_, annealed oligonucleotides #7 and #8 were cloned between PsiI and HindIII sites in pESB2.61 | This work |
| pESB6.59 | T7p-*his_6_-kfrA*_RA3_, EcoRI-SalI fragment from pAKB2.60 was cloned into pET28M | This work |
| pET28M | pET28a modified | (9) |
| pMAB18.7/8 | pUC18 with R751O_K_ | (10) |
| pMAB28.1 | pET28M-T7p-*his_6_-kfrA*_R751_ | (10) |
| pMAB30.1 | pGBT30 *tacp-kfrA*_R751_ | (10) |
| pSRA28.1 | pET28M-*his_6_-cys_3_-kfrA_RA3_* with insertion of three cysteine residues codons (^5’^TGTTGCTGC^3’^) after START codon, PCR fragment amplified on RA3 template with the use of primers #19 and #20 was cloned between EcoRI and SalI sites in pET28M | This work |
| pSRA28.2 | pET28M-*his_6_-kfrA_RA3_-cys_3_ w*ith insertion of three cysteine residues codons (^5’^TGTTGCTGC ^3’^) six codons before STOP codon, PCR fragment amplified on RA3 template with the use of primers #21 and #22 was cloned between EcoRI and SalI sites in pET28M | This work |
| pSRA28.3 | pET28M-*his_6_- cys_3-_kfrA_R751_-Δα* with insertion of three cysteine residues codons (^5’^ TGTTGCTGC ^3’^) after START codon, PCR fragment amplified on pMAB30.1 template with the use of primers #23 and #24 was digested by EcoRI and NotI, subcloned in pMAB30.1, and transferred as EcoRI-SalI fragment into pET28M | This work |
| pUC18 | ori_MB1_, Ap^R^ | (11) |
| RA3 | IncU Cm^R^ Sm^R^ Su^R^ | Hayes F. (lab collection) |
| R751 | IncP-1β, Tet^R^ | (12) |

**Table S.2** **Oligonucleotides used in this study**

| Oligonucleotides used for cloning | | |
| --- | --- | --- |
| **# 1** | *kfr*A R _R751_ | ^5’^cggtcgacTCAGGTCTTTTTGCGTCCGC^3’^ |
| **# 2** | *kfr*A F _R751_ | ^5’^cggaattc**ATG**GCGATCTCGAAGGAGCA^3’^ |
| **# 3** | *kfrA* F _RA3_ | ^5’^gcgaattc**ATG**ACCATGATTAAGCCTGA^3’^ |
| **# 4** | *kfrA* R _RA3_ | ^5’^gcgtcgacTTTACGTCAATAGATAGGGG^3’^ |
| **# 5** | OG1 | ^5’^aattcGTTGTATTGTATGTATTGTATGTATTGTATTAtaag^3’^ |
| **# 6** | OD1 | ^5’^tcgacttaTAATACAATACATACAATACATACAATACAACg^3’^ |
| **# 7** | OG2 | ^5’^AAATACAATACATACAATACAGGa^3^ |
| **# 8** | OD2 | ^5’^agcttCCTGTATTGTATGTATTGTATTT^’3’^ |
| **# 9** | OG5 | ^5’^TA*TGTATTGTATGTATTGTA*TTAAAAT*ACAATACATACAATACA*GG^3’^ |
| **# 10** | OD5 | ^5’^CC*TGTATTGTATGTATTGT*ATTTTAA*TACAATACATACAATACA*TA^3’^ |
| Oligonucleotides labeled with Cy3 or Cy5 at 5’ end and used for EMSA | | |
| **# 15** | OK_R751_  -F | ^5’^Cy5- CTTGCGTAGT*GTTGTATGTTGTAGTATG*CTA*CACGCTACAACATACAAC*C^3’^ |
| **# 16** | OK_R751_  -R | ^5’^Cy5- G*GTTGTATGTTGTAG*CG*TG*TAG*CA*TA*CTACAACATACAAC*ACTACGCAAG^3’^ |
| **# 17** | OK_RA3_-F | ^5’^Cy3-TCCGT*TGTATTGTATGTATTGTATGTATTGTA*TTAAAAT*ACAATACATACAATAC A*GGGA^3’^ |
| **# 18** | OK_RA3_  -R | ^5’^Cy3-  TCCC*TGTATTGTATGTATTGTAT*TTTA*ATACAATACATACAATACATACAATAC A*ACGGA^3’^ |
| Oligonucleotides used for Cys-labelling of N or C-end of KfrA proteins | | |
| **# 19** | EcoRI_CysN_RA3_F | ^5’^ccgcagaattc**ATG**TGTTGCTGCACCATGATTAAGCCTGA^3’^ |
| **# 20** | SalI_RA3_R | ^5’^gtatgtcgacT**TTA**CTCTTTGGTGTCGGCTGCGTCTTTTTTC^3’^ |
| **# 21** | EcoRI_KfrA_RA3_F2 | ^5’^ccgcggaattc**ATG**ACCATGATTAAGCCTGAAATGAAAGAAAAGATCGTCC^3’^ |
| **# 22** | SalI_C_wyzej_RA3_Cterm_R1 | ^5’^gtatgtcgacT**TTA**CTCTTTGGTGTCGGCTGCGCAGCAACAGTCTTTTTT^3’^ |
| **# 23** | EcoRIKfrAR751-NCCCF | ^5’^ccgcagaattc**ATG**TGTTGCTGCGCGATCTCGAAGGAGCAG^3’^ |
| **# 24** | KfrAR751-NotI-NR | ^5’^TATTATAATATCATTCGCGGCCGCCTCGACGGCCTCCAGGGC^3’^ |

Cysteine codons are highlighted in grey in the oligonucleotides sequences.

**Fig S.1. Dimerization *in vitro* of KfrA_Δ136-206_ of R751 plasmid**

The purified His-tagged KfrA protein (0.1 mg ml^-1^) was incubated with different concentrations of glutaraldehyde (GA) for 20 min at room temperature and the products were separated on 12% SDS-polyacrylamide gel. Dimers, tetramers and other higher order complexes are indicated by arrows. M- protein markers.


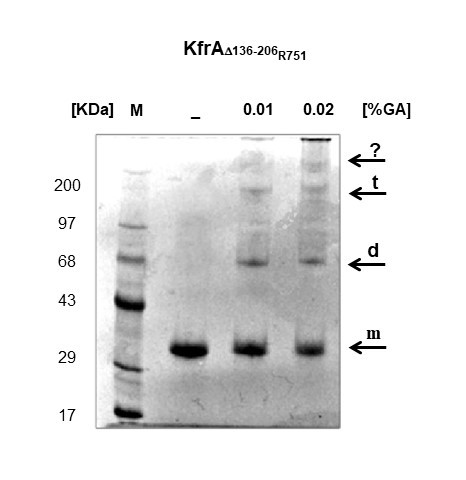


**Fig. S.2. Modeling of a control coiled-coil domain with “Fold-and-dock”.** Each point corresponds to a single model. X and Y axes define RMSD to the native structure and Rosetta energy score, respectively.


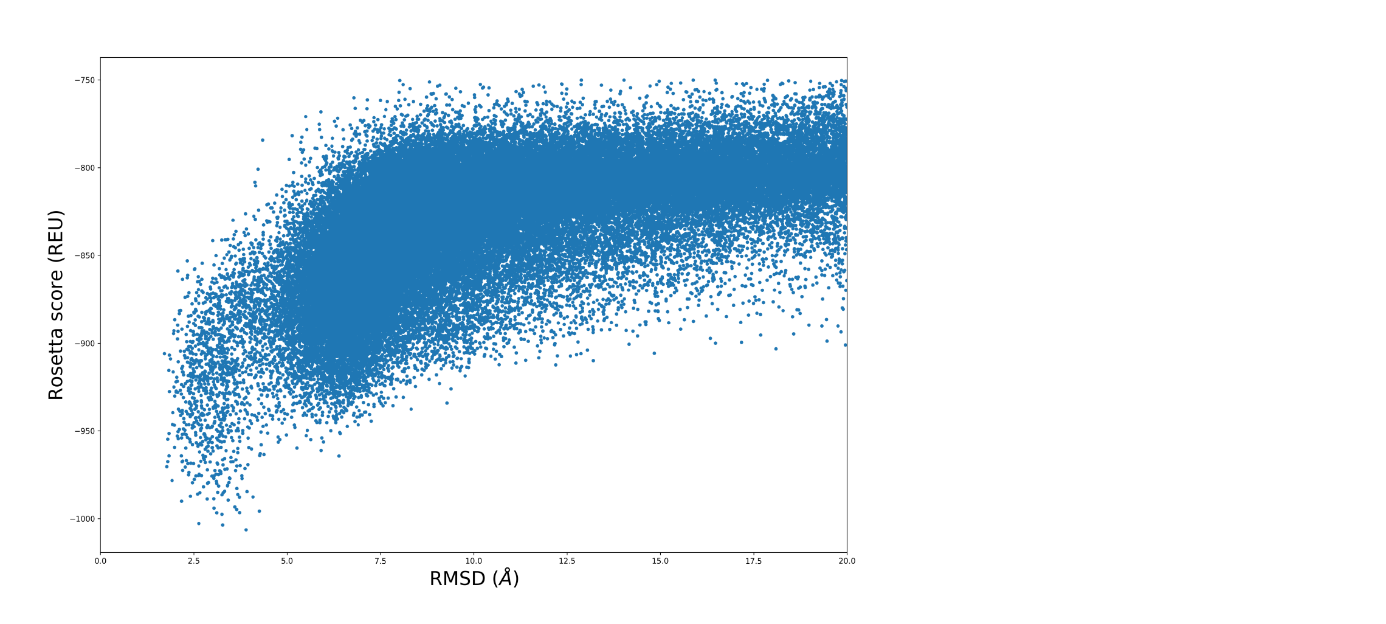


**Fig. S.3. Amino acid composition of KfrA_R751_ (residues 77-327), KfrA_RA3_ (residues 76-326), and a control coiled-coil domain.** For the definition of heptad, positions refer to the legend of Fig. 5 in the main text. Each box depicts a single heptad position in a given protein (columns): X and Y axes define side-chain volume and its abundance relative to the reference set of 800 parallel dimeric coiled-coils, respectively.


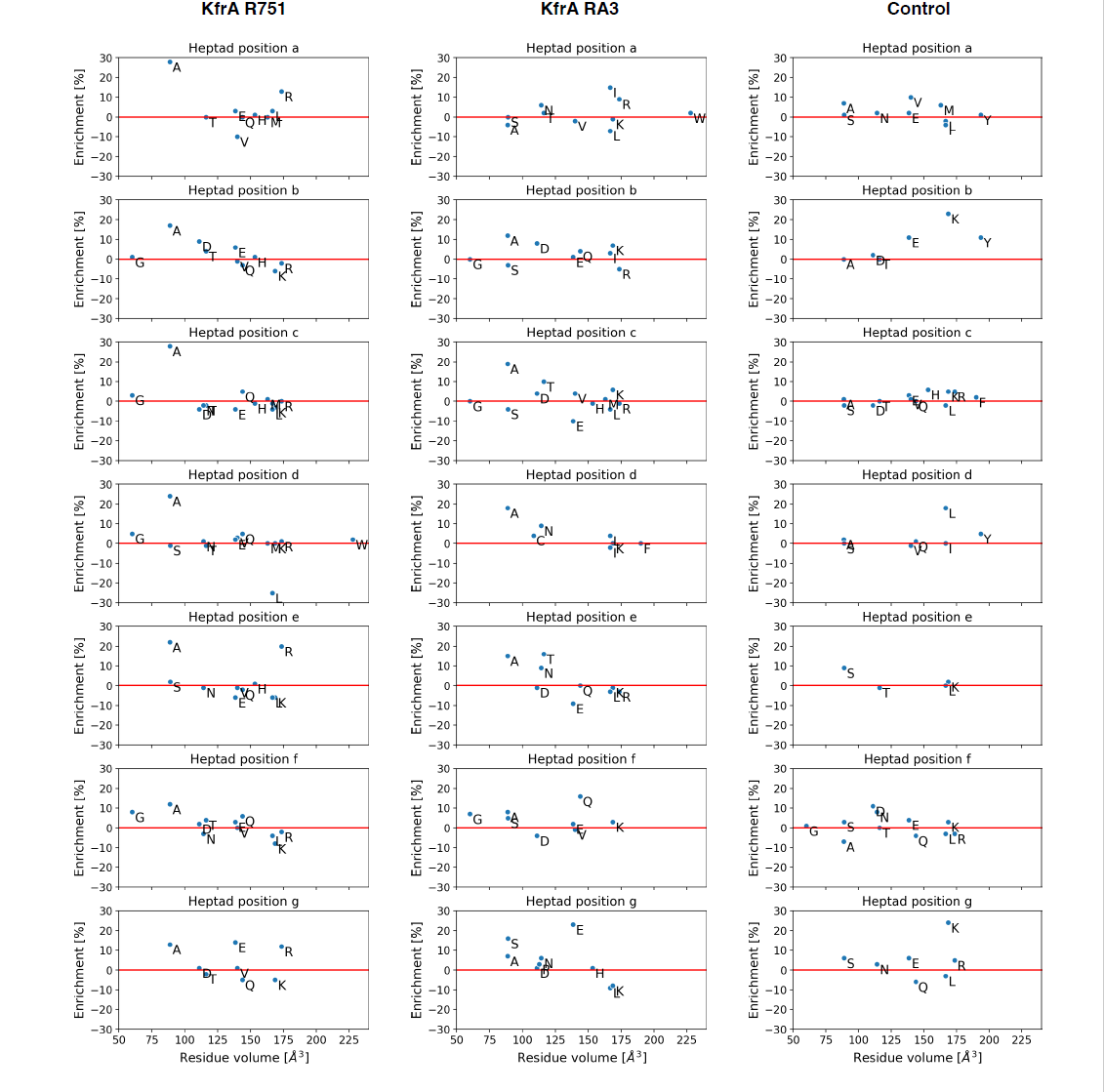


**References**

1. Jorgensen WL, Chandrasekhar J, Madura JD, Impey RW, Klein ML. Comparison of simple potential functions for simulating liquid water. J Chem Phys. 1983 Jul 15;79(2):926–35.

2. Liu DC, Nocedal J. On the limited memory BFGS method for large scale optimization. Mathematical Programming. 1989 Aug 1;45(1):503–28.

3. Nocedal J. Updating quasi-Newton matrices with limited storage. Math Comp. 1980;35(151):773–82.

4. Maier JA, Martinez C, Kasavajhala K, Wickstrom L, Hauser KE, Simmerling C. ff14SB: Improving the Accuracy of Protein Side Chain and Backbone Parameters from ff99SB. Journal of Chemical Theory and Computation. 2015;11(8):3696–713.

5. Darden T, York D, Pedersen L. Particle mesh Ewald: An N⋅log(N) method for Ewald sums in large systems. J Chem Phys. 1993 Jun 15;98(12):10089–92.

6. Ryckaert J-P, Ciccotti G, Berendsen HJC. Numerical integration of the cartesian equations of motion of a system with constraints: molecular dynamics of n-alkanes. Journal of Computational Physics. 1977 Mar 1;23(3):327–41.

7. Case DA, Ben-Shalom IY, Brozell SR, Cerutti DS, Cheatham III TE, Cruzeiro VWD, et al. Amber 2018. University of California, San Francisco. 2018.

8. Eastman P, Swails J, Chodera JD, McGibbon RT, Zhao Y, Beauchamp KA, et al. OpenMM 7: Rapid development of high performance algorithms for molecular dynamics. PLoS Computational Biology. 2017;13(7).

9. Lukaszewicz M, Kostelidou K, Bartosik AA, Cooke GD, Thomas CM, Jagura-Burdzy G. Functional dissection of the ParB homologue (KorB) from IncP-1 plasmid RK2. Nucleic Acids Res. 2002 Feb 15;30(4):1046–55.

10. Adamczyk M, Dolowy P, Jonczyk M, Thomas CM, Jagura-Burdzy G. The kfrA gene is the first in a tricistronic operon required for survival of IncP-1 plasmid R751. Microbiology (Reading, Engl). 2006 Jun;152(Pt 6):1621–37.

11. Yanisch-Perron C, Vieira J, Messing J. Improved M13 phage cloning vectors and host strains: nucleotide sequences of the M13mp18 and pUC19 vectors. Gene. 1985;33(1):103–19.

12. Thorsted PB, Shah DS, Macartney D, Kostelidou K, Thomas CM. Conservation of the genetic switch between replication and transfer genes of IncP plasmids but divergence of the replication functions which are major host-range determinants. Plasmid. 1996 Sep;36(2):95–111.
